# Effects of cumulative trauma load on long-term trajectories of life satisfaction and health in a population-based study

**DOI:** 10.1186/s12889-020-09663-9

**Published:** 2020-10-27

**Authors:** Livia Sacchi, Mariia Merzhvynska, Mareike Augsburger

**Affiliations:** Department of Psychology, Division of Psychopathology and Clinical Intervention, University of Zurich, Binzmuehlestrasse 14/17, 8050 Zurich, Switzerland

**Keywords:** Health, Life satisfaction, LVMM, LGMM, Longitudinal, Trajectories, Traumatic events

## Abstract

**Background:**

Lifetime traumatic events are known to have a detrimental long-term impact on both mental and physical health. Yet, heterogeneity in the stress response regarding well-being in adults is not well understood. This study investigates effects of cumulative trauma on latent trajectories of two indices of well-being, subjective health and life satisfaction in a large representative sample by means of latent variable modelling techniques.

**Methods:**

Data from the pairfam study wave 2–9, a longitudinal representative survey was used (*N* = 10,825). Individuals reported on lifetime trauma type exposure on wave 7 and indicated levels of life satisfaction and health at each wave. Different types of latent Variable Mixture Models were applied in an iterative fashion. Conditional models investigated effects of cumulative trauma load.

**Results:**

The best fitting model indicated three latent trajectories for life, and four for health, respectively. Trauma load significantly predicted class membership: Higher exposure was associated with non-stable trajectories for both indices but followed complex patterns of both improving and decreasing life satisfaction and health. Trauma load also explained variability within classes.

**Conclusions:**

The current study expands on evidence to the long-term development of health and life satisfaction in response to traumatic events from a latent variable modelling perspective. Besides detrimental effect, it also points to functional adaptation after initial decline and increased well-being associated with trauma exposure. Thus, response to traumatic stress is marked by great heterogeneity. Future research should focus on variables beyond exposure to trauma that can further identify individuals prone to trajectories of declining well-being.

## Introduction

Pooling data from the World Mental Health survey resulted in 70% of respondents having experienced at least one traumatic event in their lifetime, with the majority reporting multiple exposure to different types [1, 2]. In these studies, traumatic events were conceptualized as exposure to (threatened) death, injury or sexual violence, thus following the definition of the Fifth Version of Diagnostic and Statistical Manual of Mental Disorders (DSM-5; APA, 2013). Exposure to these events can be detrimental to mental and physical well-being [3] and, as a result, an increased risk for posttraumatic stress disorder (PTSD) is frequently reported [1]. For instance, in a representative German study, conditional prevalence rate for PTSD was up to 17% after exposure to sexual violence including both incidences within and outside of the family [4]. However, traumatic events also affect other important domains of well-being and are considered an unspecific risk factor for the development of health complaints [5, 6].

In the mid-90s, the so-called adverse childhood experiences (ACE) study was the first large investigation on stressful and critical life events during childhood and revealed detrimental long-term effects towards later life such as somatic complaints or mental health disturbances [7, 8]. Whilst this initial conceptualization of ACE extends beyond the diagnostic definition of trauma and thus incorporates a broader range of not necessarily traumatic events such as parental absence due to divorce (c.f [9].), the ACE study has launched the systematic investigation of long-term effects of adverse experiences. Today, findings have been confirmed by a number of recent studies, as reviews and meta-analysis pointed out (e.g., [6, 10, 11]). Crucially, since adverse events frequently co-occur due to a re-victimization cycle that is based on individual differences in liability, economic and psychological resources and life circumstances [12], dose-response models state that adversities piling up are more detrimental compared to single events. Regarding ACEs, a meta-analysis confirmed that individuals with more than four different types of exposure to ACEs were at an increased risk for worse subjective mental and physical health conditions that extended into adulthood [11].

However, when narrowing the definition of trauma exposure towards its stricter diagnostic definition and specifically focussing on the impact of cumulative trauma load across the entire life span, research is less abundant. Also, it mainly concentrates on older individuals: Lifetime trauma load was associated with decreased life satisfaction in a large nationwide study from the US [13] and another representative study found an increased risk for depressive symptoms, poorer subjective health as well as less life satisfaction [14]. With regard to physical health, traumatic experiences led to a substantial increase in the risk of suffering from common chronic diseases and medical conditions, independently from PTSD in a population-based study from Germany [15].

More recently, the long-term consequences of trauma on health have also started to be explored by data-driven approaches [16]. As a major advantage, these models allow to investigate individuals that group into distinct latent profiles over the course of time by exploring inter-individual differences in intra-individual change [17]. Thus, the novelty lies in the possibility of capturing heterogeneity in traumatic stress responses, being characterised by individuals’ idiosyncratic patterns [18]. Out of these data-driven approaches, the currently most important group of modelling techniques are Latent Variable Mixture Models (LVMM). In contrast to more traditional analyses, modelling distinct profiles of health following trauma by means of LVMMs led to important findings. A recent review cumulated evidence that the most prevalent reaction towards traumatic stress was long-term resilience as indicated by a large group of individuals with low levels of symptoms and high functionality over the course of time [16]. This resilient group was followed by much smaller groups of recovery, chronic and delayed onset [16]. This finding is important, since it also points to the possibility of functional adaptation to traumatic events. However, evidence about long-term reactions based on LVMMS are far from conclusive. Another recent systematic review cumulated evidence for a slow recovery trajectory as the most common response to stress in contrast to a resilient group [19]. The authors raised concern for previous methodological approaches in the implementation of LVMMs that could provide an explanation for this incongruence in findings [19].

Despite these methodological differences, in both reviews only a minority of studies was found to focus on well-being outcomes beyond PTSD. Thus, the majority did not consider important domains such as life-satisfaction or subjective health. Even more crucial, these few studies did not specifically investigate responses to traumatic events as defined in DSM-5, but rather focused on a single major life event. Likewise, these samples consisted of very specific populations like survivors of a major burn injury or oil rig disaster survivors that might not be indicative of the general population [16, 19]. Taken together, the heterogeneity of trajectories of well-being in response to traumatic stress is still not well understood. Even more crucial from a life course perspective, the vast majority of studies included in both reviews endorsed a pre-post adversity framework: that is, health outcomes were assessed as a temporal change before and after a single stressful event [19, 20]. However, in light of the evidence from dose-response models [13–15], it is essential to investigate how individuals group into distinct profiles of well-being over the course of time in response to multiple traumatic events. Yet, studies employing cumulative load as predictors of well-being within a LVMM framework are sparse. One single study investigated the impact of cumulative psychosocial adversities on trajectories of physical health in adolescents and found a trajectory with increasing health problems when having experienced more adversities [21]. It remains to be assessed if these patterns also exist in adulthood when applying a narrower definition of life events that only focusses on lifetime trauma load. Finally, although some studies employing a post-adversity framework exist [22, 23], evidence concerning the impact of cumulative trauma load on well-being using LVMM models within a life-span perspective, regardless of pre-post change and with a focus on the general population, is currently missing. Yet, a life-course perspective is particularly useful: having a deeper insight into the question whether well-being trajectories remain stable or undergo change over time will not only allow to ascertain the nature of their course [24] but ultimately, it could lead to intervene on them under a person-centred perspective [25].

Therefore, the aim of the current study was to explore trajectories of psychological long-term responses associated with trauma load from a life course perspective and in a large representative sample. A focus was set on two markers of overall well-being, subjective health and life satisfaction that have been frequently investigated in traditional analyses (e.g., [13–15]) but have been limitedly explored in data-driven modelling. By means of applying LVMMs as modelling technique, insights into inter- and intra-individual variability of the stress response should be gained. We hypothesized to find non-linear trajectories of both accelerating and decelerating subjective health and life satisfaction over the course of time. In line with the dose-response model [13–15], we assumed a trajectory with less stable well-being for individuals with high trauma load.

## Methods

### Participants and procedures

Analyses were based on de-identified data from the “Panel Analysis of Intimate Relationships and Family Dynamics” (*pairfam)* survey, r*elease 9.1* [26]. Pairfam is a German ongoing representative longitudinal survey about well-being and social dynamics over the life course. Beginning in 2008, data of main respondents were collected in annual waves from a random sample of three birth cohorts. Participation was voluntary and all respondents had to provide an informed consent. A detailed description is reported in Huinink et al. [27]. For the current project, data from wave 2–9 was used. Analyses were performed on all individuals above the age of 16 (*N* = 10,825). When adding predictor and covariates, analyses were conducted on *N* = 4819. At baseline, 48.3% of participants were male, with a mean of 9.3 years spent in education (*SD* = 5.98). Mean age at wave 2 was 26.43 (*SD* = 8.52), and 32.51 (*SD* = 8.32) at wave 7. Further wave-specific socio-demographic characteristics are reported in Supplementary Tables 1, 2 and 3 and Supplementary Figure 1. Regarding the attrition rate, it was found that the structure of the sample had no significant distortion, and there was no significant selectivity [28]. Ethical approval for the project was obtained from the Philosophical Faculty’s Ethics Committee of the University of Zurich.

### Measures

Measures were assessed by means of computer-assisted interviews or self-reports. Sociodemographic variables, such as age and gender, were assessed at each wave.

#### Traumatic life events

At wave 7, participants were asked to indicate if distressing life events had occurred during their life (no = 0, yes = 1). We selected all items known to be possibly traumatic: 1) Severe physical illness or accident; 2) Being a victim of a robbery or burglary; 3) Being a victim of physical violence; 4) Victim of sexual assault. Items endorsed were summed up to reflect overall traumatic load, ranging from 0 to 4. This scale was used in the LVMMs with covariates (i.e. conditional models).

#### Life satisfaction

Participants were asked to rate their life satisfaction on a 10-point Likert scale (0 = ‘very dissatisfied’, 10 = ‘very satisfied’) at all nine waves.

#### Subjective health

Participants were asked to rate their subjective health status in the previous 4 weeks on a 5-point Likert Scale. (1 = Bad; 2 = Not So Good; 3 = Satisfactory; 4 = Good; 5 = Very Good).

### Statistical analyses

Different models of LVMMs were built in an iterative process in order to derive the best model, and thus strictly following state-of-the art procedures and under missing Robust (Full Information) maximum likelihood estimation [17, 29, 30]. In the first step, single-group Latent Growth Curve Models were estimated to identify the best function representing change over time (that is linear, quadratic, cubic and latent basis). For a comparison between the quadratic and the linear model functions the nested *χ*^2^ difference test (*χ*^2^_DIFF_) was applied. A significant *χ*^2^_DIFF_ value indicates that the quadratic model fits significantly better compared to the linear model. For comparing quadratic and linear with the latent basis function, Akaike Information Criterion (AIC) and Bayesian Information Criterion (BIC) were used, with lower values indicating a better model fit. Additionally, the following fit indices were applied: standardized root mean residual (SRMR) ≤ .08; root mean square error of approximation (RMSEA) ≤ .06, comparative fit index (CFI) and the Tucker-Lewis index (TLI) ≥ .95 [29].

Subsequently, based on the best fitting function, three incremental types of models with increasing flexibility were computed (see Additional file 1): 1) A reduced model that assumes homogenous trajectories for all individuals within a class, namely Latent Class Growth Analysis (LCGA); 2) Class-Invariant Growth Mixture Models (GMM-CI) that assume equal variances and covariances across all classes; and 3) Class-varying GMMs (GMM-CV) that allow variances and covariances to vary across all classes. In order to select the best incremental model among the three, the one with the smallest Bayesian Information Criteria (BIC) value was selected [19].

Within each incremental model (LCGA, GMM-CI, GMM-CV), 1–4-class solutions were explored. To select the best fit model for each outcome, we examined multiple model fit indices, including AIC, BIC, bootstrapped likelihood ratio test (BLRT) and Lo-Mendell-Rubin likelihood ratio test (LMR-LRT), class sizes and entropy. Entropy represents the classification quality index, and it ranges from 0 and 1, with higher values indicating the classes are more easily distinguished [29]. Values of .40, .60 and .80 represent low, medium and high-class distinction respectively. The class solution with the smallest BIC and AIC, entropy values above .80, and significant BLRT and LMR-LRT are considered in the literature as optimal models [28]. In case of contradicting test results, LMR-LRT was preferred, since it is less likely to be biased [29]. Additionally, in the case of similar fit indices, the more parsimonious model with fewer but larger classes (overall sample in the smallest class > 5% and/or *n* > 25) was chosen provided that interpretability of trajectories was possible [29].

#### Conditional models

For all models, age (years) and gender (0 = Male; 1 = Female) were taken into account as covariates. In order to estimate associations between trauma load and class membership, and therefore between-class variation, group membership for both Life Satisfaction and Health was regressed on the aforementioned variables in a logistic regression fashion using the one-step approach [29]. The resulting odds-ratio (OR) describes individuals’ change in odds to be in the respective latent trajectory compared to the reference for one unit increase in trauma load and in age, and for being male versus female. Additionally, if the models allowed, mixture regression analyses were run to explore variation within each class (class specific intercept, slope and quadratic terms). Analyses were carried out within the R environment, version 3.6.1 [31] and using Mplus, version 8.1 [32].

## Results

### Trajectories of life satisfaction

#### Life satisfaction model function and best-fitting model

The linear, quadratic and the latent basis functions were successfully fitted, while the cubic function did not converge. A significant *χ*^2^_DIFF_ test as well as lower AIC indicated that the quadratic model should be preferred (see Supplementary Table 4). Accordingly, the quadratic function was further used to fit LVMM models. The quadratic term accounts for nonlinear trajectories and represents the average accelerating or decelerating change in participants’ trajectories over time [29]. Comparing the quadratic GMM-CI models with the quadratic LCGA models (see Supplementary Table 5), across all four class-solutions, the former had lower BIC values. For GMM-CV, model building errors were consistently occurring and thus the overall GMM-CV model had to be discarded. Accordingly, the GMM-CI was considered the best model.

#### Selection of number of classes

BIC was lowest for the 4-class solution, followed by the 3-class solution. Entropy values for both classes were above .80. However, the LMR-LRT was only significant for the 3-class solution. Thus, the 3-class model was chosen as final one. Fit indices for GMM-CI with all class-solutions are reported in Table 1. All model coefficients of the 3-class solution are reported in Supplementary Table 6.

**Table 1:** Fit Statistics of the 1–4 class solutions for Life Satisfaction Quadratic GMM-CI

	1 Class	2 Classes	3 Classes	4 Classes
LL (No. of parameters)	−99,013.54 (17)	−98,290.23 (21)	− 97,849.11 (25)	− 97,563.94 (29)
AIC	198,061.08	196,622.45	195,748.23	195,185.89
BIC	198,185.01	196,775.53	195,930.46	195,397.28
Entropy	N/A	0.875	0.848	0.840
LMR-LRT	N/A	1408.72***	859.109**	555.39
BLRT	N/A	1446.63***	859.109***	570.34***
Group-size (%) Class 1	10,824 (100%)	9974 (92.1%)	363 (3.3%)	447 (4.1%)
Class 2	N/A	850 (7.9%)	560 (5.2%)	354 (3.3%)
Class 3	N/A	N/A	9901 (91.5%)	272 (2.5%)
Class 4	N/A	N/A	N/A	9751 (90.1%)

*Note: GMM-CI* Growth Mixture Model with class-invariant variances and covariances, *LL* Log-Likelihood value, *No. of Parameters* Number of estimated parameters, *AIC* Akaike Information Criterion, *BIC* Bayesian Information Criterion, *LMR-LRT* Lo-Mendell-Rubin Likelihood Ratio Test, *BLRT* Bootstrap Likelihood Ratio Test

**p < .01

***p < .001

**Fig. 1:**
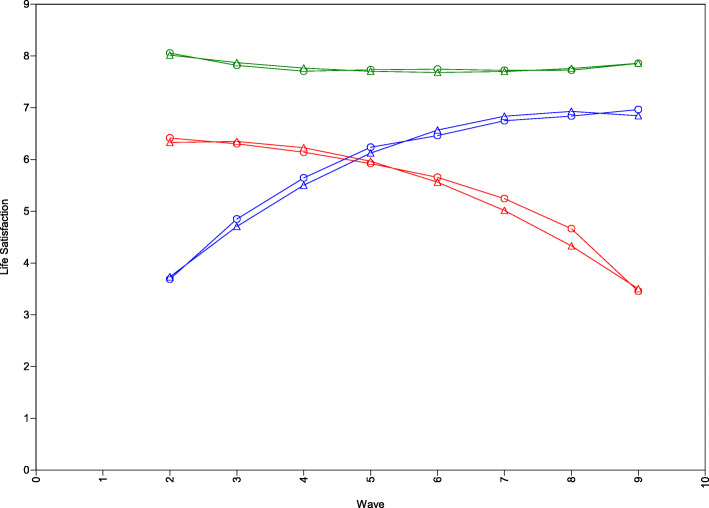
Latent Trajectories for the GMM-CI 3-class solution for Life Satisfaction. The red trajectory represents class 1 (“Stable class”), the blue trajectory represents class 2 (“Declining class”), and the green trajectory represents class 3 (“Improving class”). Circles refer to sample means; triangles refer to estimated means

The quadratic 3-class GMM-CI solution points to the existence of a smaller class of individuals with high levels of Life Satisfaction that declined over time (3.3%, Class 1, red trajectory, “Declining class”) and another small class of individuals with initially low levels of life satisfaction which markedly improved across the years (5.2%; Class 2, blue trajectory, “Improving class”). Finally, there is a large class of individuals (91.5%; Class 3, green trajectory) with high and stable levels of Life Satisfaction over the course of time (“Stable class”). The trajectories are visually depicted in Fig. 1.

#### Conditional model

Cumulative trauma load emerged as a significant correlate of class membership. With increasing exposure to traumatic events, individuals were both more likely to be in the declining class and also more likely to be in the improving class, both compared to the stable class. Although significant, age was not heavily related to class membership of the improving class. Within-class variation indicated that increasing exposure to traumatic events was associated with significantly lower intercept scores in the declining class, and with lower slope levels in the stable class, while also positively affecting the quadratic term. By contrast, there was no within-class variation in the improving class. Age was associated with significantly lower intercept scores and with positive quadratic term scores in the stable class. All coefficients are reported in Table 2.

**Table 2:** Conditional Model for Life Satisfaction

Classes	Cumulative Trauma			Sex (Male vs Female)			Age		
Between-class (multinomial regression coefficients)	Estimate	S.E	OR [95% CI]	Estimate	S.E.	OR [95% CI]	Estimate	S.E.	OR [95% CI]
Improving vs Stable^a^	0.563*	0.244	1.76 [1.04; 2.83]	0.129	0.240	1.14 [1.41; 1.82]	0.046***	0.012	1.05 [0.97; 1.07]
Declining vs Stable^a^	0.558**	0.204	1.75 [1.17; 2.60]	−0.047	0.210	0.95 [0.63; 1.44]	−0.004	0.012	0.99 [0.97; 1.02]
**Within-class (mixture regression coefficients)**									
Improving (3.3%)									
Intercept	0.126	0.235		0.236	0.326		−0.005	0.020	
Slope	−0.176	0.131		0.141	0.222		−0.016	0.013	
Quadratic term	0.025	0.025		−0.011	0.028		0.001	0.002	
Declining (5.2%)									
Intercept	−0.387*	0.158		−0.680*	0.335		−0.041	0.023	
Slope	0.074	0.093		0.195	0.192		0.001	0.012	
Quadratic term	−0.001	0.010		−0.013	0.027		−0.001	0.002	
Stable (91.5%)									
Intercept	−0.036	0.042		0.021	0.045		−0.013***	0.002	
Slope	−0.115***	0.025		−0.034	0.021		0.003**	0.001	
Quadratic term	0.015***	0.003		0.006*	0.003		0.000	0.000	

*Note: S.E.* Standard Error, *OR* Odds Ratio, *CI* Confidence Intervals

*p < .05

**p < .01

***p < .001

^a^Stable Life Satisfaction is the reference class

### Trajectories of health

#### Health model function and best-fitting model

Again, the quadratic model was preferred over the linear and the latent basis model (see Supplementary Table 7), whilst the cubic function did not converge. Regarding the GMM-CI model, a constrained model had to be implemented (variance set to 0) due to model building errors. The constrained GMM-CI models reported lower BIC values than the LCGA models (see Supplementary Table 8). GMM-CV model was discarded due to the convergence error. Thus GMM-CI models were preferred.

#### Selection of number of classes

The 4-class constrained GMM-CI model had higher entropy levels for larger classes solutions, as well as lower BIC and AIC values, compared to the other solutions. Both LMR-LRT and BLRT tests were significant. Accordingly, GMM-CI with 4 classes was retained as the final model. Details are reported in Table 3. All model coefficients of the 4-class solution are reported in Supplementary Table 9.

**Table 3:** Fit Statistics of the 1–4 class solutions for Health constrained Quadratic GMM-CI

	1 Class	2 Classes	3 Classes	4 Classes
LL (NO. Of parameters)	−72,988.13 (14)	−72,732.27 (18)	−72,531.49 (22)	−72,419.29 (26)
AIC	146,004.25	145,500.55	145,106.98	144,890.57
BIC	146,106.30	145,631.76	145,267.36	145,080.09
Entropy	N/A	0.642	0.660	0.676
LMR-LRT (*p*-value)	N/A	498.29***	391.03*	218.54***
BLRT (*p*-value)	N/A	511.71***	401.56***	224.42***
Group-size (%) Class 1	10,822 (100%)	1752 (16.2%)	761 (7%)	1243 (11.4%)
Class 2	N/A	9070 (83.8%)	8697 (80.4%)	559 (5.2%)
Class 3	N/A	N/A	1364 (12.6%)	377 (3.5%)
Class 4	N/A	N/A	N/A	8643 (79.9%)

*Note: GMM-CI* Growth Mixture Model with class-invariant variances and covariances, *LL* Log-Likelihood value, *No. of Parameters* Number of estimated parameters, *AIC* Akaike Information Criterion, *BIC* Bayesian Information Criterion, *LMR-LRT* Lo-Mendell-Rubin Likelihood Ratio Test, *BLRT* Bootstrap Likelihood Ratio Test

*p < .05

***p < .001

**Fig. 2:**
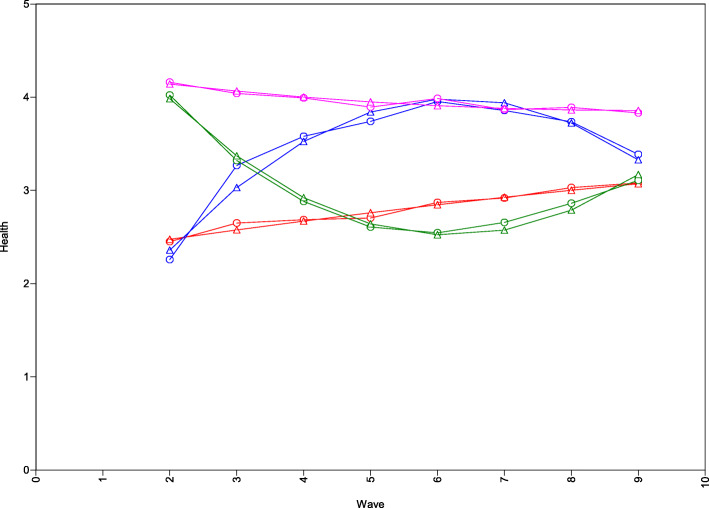
Latent Trajectories for the constrained GMM-CI 4-class solution for Health. The red trajectory represents class 1 (“Improve-decline class”), the blue trajectory represents class 2 (“Decline-low recovery”), the green trajectory represents class 3 (“Improving class”), and the magenta trajectory represents class 4 (“Stable class”). Circles refer to sample means; triangles refer to estimated means

The 4-class solution pointed to two smaller classes of individuals with lower starting levels of health and two with higher levels. Health of individuals in Class 1 (11.4%) followed a linear trajectory of slight improvement across the years (red trajectory, “Improving class”). Health of individuals in Class 2 (5.2%) followed an inversed U-shaped development with an initial improvement until a peak and subsequent decline (blue trajectory, “Improve-decline class”). Class 3 (3.5%) showed the reversed pattern with higher health at the beginning, decrease in the following years until reaching the lower point around Wave 5 and slightly increase after (green trajectory, “Decline-low recovery class”). Finally, Class 4 (79.9%) comprised the majority of individuals whose high level of health remained relatively stable across the years (pink trajectory, “Stable class”). All trajectories are visually depicted in Fig. 2.

#### Conditional model of health

Cumulative trauma load also emerged as a significant correlate of class membership. With increasing exposure, compared to the stable class, individuals were more likely to be in improve-decline class, followed by the improving health class and the decline-low recovery health class. The two covariates were also significantly associated with class membership: individuals belonging to the decline-low recovery and to the improving classes were more likely to be females and of younger age. Regarding within-class variations, increasing trauma load was related to a lower intercept score and to a positive quadratic term score in the decline-low recovery class. Similarly, increasing exposure was associated with a positive quadratic term in the improve-decline class. Overall, female gender and age were significantly associated with all within-class parameters. All coefficients are presented in Table 4.

**Table 4:** Conditional Model for Health

Classes	Cumulative Trauma			Sex (Male vs Female)			Age		
Between-class (multinomial regression coefficients)	Estimate	S.E	OR [95% CI]	Estimate	S.E.	OR [95% CI]	Estimate	S.E.	OR [95% CI]
Decline-low recovery vs Stable^a^	0.702**	0.206	2.018 [1.35; 3.02]	0.648*	0.278	1.912 [1.11; 3.29]	− 0.039**	0.011	0.962 [0.94; 0.98]
Improving vs Stable^a^	0.703**	0.217	2.019 [1.32; 3.09]	0.756*	0.334	2.130 [1.11; 4.09]	−0.054**	0.017	0.948 [0.92; 0.98]
Improve-decline vs Stable^a^	0.917***	0.195	2.502 [1.71; 3.67]	−0.567	0.341	0.567 [0.29; 1.11]	−0.007	0.017	0.993 [0.71; 1.39]
**Within-class (mixture regression coefficients)**									
Decline-low recovery (12.3%)									
Intercept	0.037	0.070		−1.224***	0.184		−0.020**	0.008	
Slope	−0.118**	0.042		0.682***	0.110		0.002	0.005	
Quadratic term	0.012*	0.005		−0.068***	0.014		0.000	0.001	
Improving (7%)									
Intercept	−0.102	0.112		−0.460*	0.179		−0.013	0.011	
Slope	0.023	0.059		−0.422***	0.114		−0.016*	0.007	
Quadratic term	−0.006	0.008		0.072***	0.017		0.003*	0.001	
Improve-decline (6.3%)									
Intercept	−0.043	0.117		0.638	0.352		0.001	0.014	
Slope	−0.102	0.067		0.253	0.148		−0.015*	0.007	
Quadratic term	0.018*	0.008		−0.060**	0.019		0.001	0.001	
Stable (74.4%)									
Intercept	−0.049	0.041		−0.057	0.035		−0.016	0.002	
Slope	−0.036	0.026		−0.055**	0.021		0.003**	0.001	
Quadratic term	0.005	0.004		0.008**	0.003		0.000	0.000	

*Note: S.E.* Standard Error, *OR* Odds Ratio, *CI* Confidence Intervals

*p < .05

**p < .01

***p < .001

^a^Stable Health is the reference class

## Discussion

Despite the surge in LVMM applications in the field of traumatic stress, no studies have investigated the effects of lifetime cumulative trauma load on longitudinal trajectories of subjective well-being such as life satisfaction and health*.* To address this gap, two indices of overall well-being, namely life satisfaction and subjective health were investigated in a large representative sample over the course of time based on a flexible modelling approach that could account for both inter- and intra-class variability. As a result, modelling trajectories led to a 3-class solution for life satisfaction, and a 4-class solution for health, respectively. For both indices, the majority of individuals were grouped within trajectories of relatively stable high levels. Likewise, a trajectory with initially low values but consistently improving health and life satisfaction was observed. For life satisfaction, the third group consisted of individuals with consistently declining life satisfaction over the course of time. For health, two more classes were apparent: One group with an increase of health until it reached a peak in health and a subsequent decline, and a one with a reversed U-shaped pattern of initial decline followed by late recovery. Regarding associations with cumulative trauma load on well-being, it was found to differentiate between the classes. Individuals with increasing trauma load were more likely not to be in the stable trajectories, but in all other trajectory classes. Moreover, cumulative trauma partially explained within-class differences in both life satisfaction and health.

This finding contributes to the evidence that exposure to trauma has a long-term impact on well-being. The current results partially align with previous studies that point to detrimental effects of victimization in both older and younger populations by investigating linear associations between these variables [13–15, 33, 34]. However, the current findings go beyond models assuming linear associations, but support more complex patterns that might not become visible without latent variable modelling techniques. Moreover, they enhance previous conclusions from the two reviews on adaptations to stressful life events that were mainly based on a diagnostic classification such as PTSD and/or on very specific trauma types and samples [16, 19]. For health we found empirical support for a group of individuals that experience a decrease, followed by partial recovery. This is accordance with the concept about a dynamic phase of adaptation as the most prominent reaction [19]. Thus, cumulative trauma exposure might be associated with both a detrimental effect on well-being and an eventually positive development. The current study demonstrates that adaptations to traumatic events might be marked by inter-individual variability, resulting in substantial heterogeneity of individuals’ profiles [19, 35]. Further supporting this view, within-class variation showed that cumulative trauma was negatively related to the rate of change and the accelerating change of the decline-low recovery class, and similarly the improve-decline class. Depending on the specific class, individuals with higher trauma exposure reported initial lower subjective health or less subjective health over the course of time.

A similar finding was also observed within the decreasing class of life satisfaction and thus aligns with previous results reported in adolescent samples [21]. Again, these results argue in favour that more trauma exposure increases the risk for an interruption of normal life and requires fundamental adaptation that might turn out to be successful. In addition, for both indices, trajectories of slowly improving well-being were found. Here, trauma load was not associated with individual within-class differences in the initial evaluation of life satisfaction and health. Thus, it is likely that other factors that were not taken into account might contribute to increased life satisfaction and subjective health associated with higher trauma load. This could be availability of resources such as coping strategies or meaningful integration of adverse experiences into the one’s life. Moreover, these classes might represent a small share of the population whose well-being improves with time following adversity. This aligns with the idea of a “steeling effect”, indicating that people exposed to a moderate amount of adversities have a higher well-being compared to individuals without adverse experiences [36].

It is important to keep in mind that interpretations are speculative and warrant further research regarding cause-effect relationships. In the current study traumatic events were not assessed at a specific time but explored throughout the lifetime. We cannot rule out that traumatic experiences have occurred after changes in trajectories on well-being. Hence it is not conclusive what exactly caused the change in trajectories at the specific time points. This is a major difference with respect to studies applying a pre/post adversity framework. Whilst important associations with trauma load were found, it cannot be conclusively stated at which time frame it had the largest impact. Crucially, causal relations between alterations in indices of well-being as a result of increasing trauma load cannot be assumed with the current study.

Nevertheless, the current study provides a number of important implications. First, it focusses on a large representative sample and thus supports the generalizability of prototypical patterns of phenotypic adaptations to stress. Moreover, the findings indicate that it is essential to not only consider a single traumatic events but incorporate the cumulative effect of different types of adversities in order to reflect experiences in real life more accurately. In addition, it demonstrates the importance of considering effects of trauma exposure on well-being beyond a diagnostic classification such as PTSD. And finally, the project indicates that changes in well-being might only be visible from a long-term perspective, and when applying latent modelling techniques. Accordingly, it is vital to incorporate large observational windows. To sum up the current study complements previous findings about long-term effects of ACEs on health towards exposure in adulthood (e.g., [6, 10, 11]) while expanding this strand of research by employing LVMM to explore the heterogeneous nature of subjective health and life satisfaction over time.

Regarding future research, these aforementioned aspects should be considered. Furthermore, in order to model the direct effect of cumulative trauma exposure, future studies should apply a time-specific framework that allows to measure time and duration of exposures. In doing so, it could be further explored whether trauma load causes a transition from a high level of life satisfaction or health to a lower level within classes. Additionally, it might be important to investigate how time-specific trauma load is linked with the onset of mental disorders from a latent trajectory modelling perspective.

And finally, knowing that cumulative trauma load differentially impacts class membership both in subjective health and in life satisfaction is also a relevant information for mental health professionals. It demonstrates the importance of a routine screening for traumatic event exposure in clinical settings. The current research undermines their relevance for well-being and this information might be important in terms of providing tailored interventions. Besides the potentially devastating effects on well-being, it is also important to consider that adaptations are marked by great inter-individual variability.

### Limitations

The current study presents several important limitations. Other potentially relevant covariates (e.g. personality, income) could not be included in the conditional models due to methodological constraints. Adding more covariates known to possibly influence the formation of the latent trajectories extracted. Thus, participants could “shift” from one class to another due to covariates. This is especially true when entropy values are low [28]. Given the optimal value of life satisfaction entropy and the low/medium quality value for health, it was decided to proceed with the addition of essential covariates only (i.e. gender and sex). In addition, the study focusses on associations with trauma load and therefore neglects other variables that might play an important role for both trauma exposure and outcomes of well-being (e.g. genetics). This further weakens the interpretation of causal effects and might affect the robustness of associations. As stated above, subjective health 4-class unconditional model presented a borderline entropy value of .67. Low/medium quality values argue that classes could not be optimally differentiated. In addition, the number of different trauma types included in the trauma load index was limited. Standard trauma event lists such as the Life Event Checklist for DSM-5 [37] cover a much broader range of potentially traumatic events. In the current study, exposure was restricted to a few items. It is likely that associations might be different when including more trauma types. Moreover, traumatic events could have experienced more than once, which was also not measured. It is possible that trauma exposure is undercounted. This was due to the pairfam study design. As a consequence, our study has a pilot character that points to the relevance of traumatic load even with a small number of traumatic experiences for overall subjective health and life satisfaction but needs to be elaborated in samples with more comprehensive measures. In addition, the time of the traumatic event should be assessed in order to confirm cause-effect relationships. Moreover, we cannot completely rule out the possibility that class membership might have changed for the conditional models. Including trauma load resulted in a different sample size, and thus a direct comparison between the unconditional and conditional models was not possible. However, since parameters had comparable values, model interpretation as we did is still warranted. Future studies could address this limitation by using imputation techniques, which however are computationally intense. Finally, the employment of a time-invariant variable such as cumulative trauma load as a predictor of life satisfaction and health membership is novel in the LVMM and traumatic stress fields. Using trauma load at wave 7 as a predictor for the conditional models is controversial, and time-invariant covariates are often used at baseline. Therefore, such approach warrants more research supporting its utility and feasibility to investigate the impact of lifetime trauma load on well-being, beyond the pre-post adversity framework.

## Conclusion

This study expands the current evidence on the long-term development of health and life satisfaction in representative samples over the course of time, in particular related to traumatic events. Besides potentially devastating effects of cumulative trauma load, it points to trajectories of functional adaptation after initial decline or even increasing well-being. Future research should focus on more specific predictors beyond trauma load such as to availability and usage of resources in order to differentiate between individuals with less favourable patterns of development that might be in need of professional support.

## Supplementary information

**Additional file 1: Table S1.** Demographics wave by wave. **Table S2.** Change in Health over the time. **Table S3.** Change in Life satisfaction over the time. **Figure S1.** Cumulative Critical Life Events Frequencies Plot. **Table S4.** Fit Statistics for Life Satisfaction Single-Group (Nonmixture) Models. **Table S5.** Fit Statistics for Life Satisfaction Quadratic LCGA. **Table S6.** Model Results of the Life Satisfaction Quadratic GMM-CI for the 3-class solution. **Table S7.** Fit Statistics for Health Single-Group (Nonmixture) Models. **Table S8.** Fit Statistics for Health LCGA. **Table S9.** Model Results of the Health Constrained Quadratic GMM-CI for the 4-class Solution

## Data Availability

The data analysed during the current study are not publicly available due to a restricted use licence. Datasets are available from GESIS (https://www.pairfam.de/en/data/data-access/) for scientific use.

## References

[1] Kessler RC, Aguilar-Gaxiola S, Alonso J, Benjet C, Bromet EJ, Cardoso G (2017). Trauma and PTSD in the WHO World Mental Health Surveys. Eur J Psychotraumatol.

[2] Benjet C, Bromet E, Karam EG, Kessler RC, McLaughlin KA, Ruscio AM (2016). The epidemiology of traumatic event exposure worldwide: results from the world mental health survey consortium. Psychol Med.

[3] Mancini AD, Bonanno GA (2010). Resilience to Potential Trauma. Toward a Lifespan Approach.

[4] Maercker A, Hecker T, Augsburger M, Kliem S (2018). ICD-11 prevalence rates of posttraumatic stress disorder and complex posttraumatic stress disorder in a German Nationwide sample. J Nerv Ment Dis.

[5] Thoits PA (2010). Stress and Health: Major Findings and Policy Implications. J Health Soc Behav.

[6] Maniglio R (2009). The impact of child sexual abuse on health: a systematic review of reviews. Clin Psychol Rev.

[7] Dube SR, Anda RF, Felitti VJ, Chapman DP, Williamson DF, Giles WH (2001). Childhood abuse, household dysfunction, and the risk of attempted suicide throughout the life span: findings from the adverse childhood experiences study. JAMA..

[8] Anda RF, Felitti VJ, Bremner JD, Walker JD, Whitfield C, Perry BD (2006). The enduring effects of abuse and related adverse experiences in childhood: a convergence of evidence from neurobiology and epidemiology. Eur Arch Psychiatry Clin Neurosci.

[9] Maercker A, Augsburger M (2019). Developments in Psychotraumatology: a conceptual, biological, and cultural update. Clin Psychol Eur.

[10] Nemeroff CB (2016). Paradise lost: the neurobiological and clinical consequences of child abuse and neglect. Neuron..

[11] Hughes K, Bellis MA, Hardcastle KA, Sethi D, Butchart A, Mikton C (2017). The effect of multiple adverse childhood experiences on health: a systematic review and meta-analysis. Lancet Public Health.

[12] Kessler RC, Aguilar-gaxiola S, Alonso J, Benjet C, Bromet EJ, Cardoso G (2017). Trauma and PTSD in the WHO World Mental Health Surveys.

[13] Krause N (2004). Lifetime trauma, emotional support, and life satisfaction among older adults. Gerontologist..

[14] Yang MS, Hedeker D (2019). A life-span approach to examining older vulnerable population’s subjective well-being: the role of adversity and trauma. Aging Ment Health.

[15] Glaesmer H, Brähler E, Gündel H, Riedel-Heller SG (2011). The association of traumatic experiences and posttraumatic stress disorder with physical morbidity in old age: a german population-based study. Psychosom Med.

[16] Galatzer-Levy IR, Huang SH, Bonanno GA (2018). Trajectories of resilience and dysfunction following potential trauma : A review and statistical evaluation. Clin Psychol Rev.

[17] Ram N, Grimm KJ (2009). Methods and measures: growth mixture modeling: a method for identifying differences in longitudinal change among unobserved groups. Int J Behav Dev.

[18] Jung T, Wickrama KAS (2008). An introduction to latent class growth analysis and growth mixture modeling. Soc Personal Psychol Compass.

[19] Infurna FJ, Luthar SS (2018). Re-evaluating the notion that resilience is commonplace: a review and distillation of directions for future research, practice, and policy. Clin Psychol Rev.

[20] Bonanno GA, Romero SA, Klein SI (2015). The temporal elements of psychological resilience: an integrative framework for the study of individuals, families, and communities. Psychol Inq.

[21] Whalen DJ, Belden AC, Tillman R, Barch DM, Luby JL (2016). Early adversity, Psychopathology, and Latent Class Profiles of Global Physical Health From Preschool Through Early Adolescence. Psychosom Med.

[22] Bonanno GA, Galea S, Bucciarelli A, Vlahov D. What predicts psychological resilience after disaster? The Role of Demographics, Resources, and Life Stress. 2007;75(5):671–82. 10.1037/0022-006X.75.5.671 17907849

[23] Zautra AJ, Johnson LM, Davis MC. Positive Affect as a Source of Resilience for Women in Chronic Pain. J Consult Clin Psychol. 2005;73(2):212–20. 10.1037/0022-006X.73.2.212. 10.1037/0022-006X.73.2.212 PMC2593933 15796628

[24] Hoekstra T, Barbosa-Leiker C, Koppes LLJ, Twisk JW. Developmental trajectories of body mass index throughout the life course: an application of Latent Class Growth (Mixture) Modelling. Longitud Life Course Stud. 2011;2(3):319-30.

[25] Henly SJ, Wyman JF, Findorff MJ (2011). Health and illness over time: the trajectory perspective in nursing science. Nurs Res.

[26] Brüderl J, Drobnič S, Hank K, Huinink J, Nauck B, Neyer FJ, Wilhelm B (2018). Beziehungs- und Familienpanel (pairfam) [Data set].

[27] Huinink J, Brüderl J, Nauck B, Walper S (2011). Panel analysis of intimate relationships and family dynamics (pairfam): conceptual framework and design. J Fam Res.

[28] Müller B, Castiglioni L, Schupp J, Wolf C (2015). Attrition im Beziehungs- und Familienpanel pairfam. Nonresponse Bias Schriftenreihe der ASI - Arbeitsgemeinschaft Sozialwissenschaftlicher Institute.

[29] Wickrama KAS, Lee TK, O’Neal CW, Lorenz FO (2016). Higher-order growth curves and mixture modeling with Mplus: A practical guide.

[30] Grimm KJ, Ram N, Estabrook R, Little TD (2016). Growth modeling: structural equation and multilevel modeling approaches.

[31] R Core Team. R: A language and environment for statistical computing. Vienna: R Foundation for Statistical Computing; 2019.

[32] Muthén LK, Muthén BO (2017). Mplus User’s guide.

[33] Boynton-Jarrett R, Ryan LM, Berkman LF, Wright RJ. Cumulative Violence Exposure and Self-Rated Health: Longitudinal Study of Adolescents in the United States. Pediatrics. 2008;122(5):16–9. 10.1542/peds.2007-3063 PMC8679309 18977974

[34] Cleland C, Kearns A, Tannahill C, Ellaway A. The impact of life events on adult physical and mental health and well - being: longitudinal analysis using the GoWell health and well - being survey. BMC Res Notes. 2016:1–9. 10.1186/s13104-016-2278-x PMC5070029 27760568

[35] Keinan G, Shrira A, Shmotkin D (2012). The association between cumulative adversity and mental health: considering dose and primary focus of adversity. Qual Life Res.

[36] Seery MD, Holman EA, Silver RC (2010). Whatever does not kill us: cumulative lifetime adversity, vulnerability, and resilience. J Pers Soc Psychol.

[37] Weathers FW, Blake DD, Schnurr PP, Kaloupek DG, Marx BP, Keane TM (2013). The Life Events Checklist for DSM-5 (LEC-5).

