# Supplementary material for: Effects of cumulative trauma load on long-term trajectories of life satisfaction and health in a population-based study
Source: BMC Public Health. 2020 Oct 27;20:1612. doi: 10.1186/s12889-020-09663-9 (PMC7590721; doi:10.1186/s12889-020-09663-9)
Supplement: Supplementary file 1 — Additional file 1: Table S1. Demographics wave by wave. Table S2. Change in Health over the time. Table S3. Change in Life satisfaction over the time. Figure S1. Cumulative Critical Life Events Frequencies Plot. Table S4. Fit Statistics for Life Satisfaction Single-Group (Nonmixture) Models. Table S5. Fit Statistics for Life Satisfaction Quadratic LCGA. Table S6. Model Results of the Life Satisfaction Quadratic GMM-CI for the 3-class solution. Table S7. Fit Statistics for Health Single-Group (Nonmixture) Models. Table S8. Fit Statistics for Health LCGA. Table S9. Model Results of the Health Constrained Quadratic GMM-CI for the 4-class Solution [file 12889_2020_9663_MOESM1_ESM.docx]

**Effects of Cumulative Trauma Load on Long-Term Trajectories of Life Satisfaction and Health in a Population-Based Study**

**- Additional File 1-**

Methods S1. *Three incremental types of modelling: LCGA, GMM-CI and GMM-CV*

Latent Class Growth Analysis (LCGA) and Growth Mixture Modelling (GMM) are part of LVMM, but are conceptually different. LCGA can be considered as a simplified version of GMM: the holding principles of LCGA are homogeneity of variance and normal distribution of data. In other words, once divided into different classes, individuals are not allowed to vary within the class they have been assigned, but instead they are forced to follow an equal pattern within the class. Their variances of the slope and intercept are therefore set to 0, while the means are allowed to vary across classes (1). In other words, people are homogeneous in their growth trajectories within their classes of belonging. According to some authors (2), this assumption is seldom tenable. All in all, LCGA present some advantages, such as that is works well with small sample sizes and when complex models fail to converge or are out of range estimates; and finally, as an initial modelling step prior to GMM specification. GMM is more versatile compared to LCGA, as it allows free estimation of different growth parameters combinations. In GMM, subjects’ growth trajectories are allowed to vary not only between classes, but also within classes, along with slopes, intercepts, covariances, residual variances and residuals. GMM is believed to be more suitable when from a theoretical perspective heterogeneity among classes is expected. However, in particular, GMM-CV is computationally burdensome: therefore, convergence problems are often reported.

The three different incremental types of models were embedded in a linear coding of time, following a life-course approach. The coding of time is an essential part of every LVMM model, and it is related to the different research questions and the data itself (3). For this specific investigation, the time-coding scheme used is as follows: 0, 1, 2, 3, 4, 5, 6, 7.  This time-coding scheme was selected based on the annual data collection as in the *pairfam.*

Table S1**.** *Demographics wave by wave*

| Wave [N] | Gender | |  |
| --- | --- | --- | --- |
|  | n male | n female | Age M, (SD) [min; max] |
| Wave 2 [9031] | 4358 | 4671 | 26.43 (8.52) [16; 39] |
| Wave 3 [9074] | 4361 | 4712 | 28.04 (8.37) [16; 40] |
| Wave 4 [8073] | 3840 | 4231 | 29.19 (8.39) [17; 41] |
| Wave 5 [7248] | 3435 | 3811 | 30.25 (8.39) [18; 42] |
| Wave 6 [6574] | 3088 | 3484 | 31.35 (8.34) [19; 43] |
| Wave 7 [5919] | 2769 | 3148 | 32.50 (8.32) [20; 44] |
| Wave 8 [5461] | 2527 | 2933 | 33.53 (8.31) [21; 45] |
| Wave 9 [5127] | 2379 | 2748 | 34.59 (8.28) [22; 46] |

Table S2**.** *Change in Health over the time*

| Name | Sample Size | Mean | Variance |
| --- | --- | --- | --- |
| Health_2 | 7376 | 3.787 | 1.056 |
| Health_3 | 9070 | 3.757 | 0.97 |
| Health_4 | 7681 | 3.717 | 0.949 |
| Health_5 | 6959 | 3.644 | 0.913 |
| Health_6 | 6321 | 3.743 | 0.967 |
| Health_7 | 5705 | 3.665 | 0.922 |
| Health_8 | 5260 | 3.700 | 0.937 |
| Health_9 | 4940 | 3.652 | 0.899 |

Table S3**.** *Change in Life satisfaction over the time*

| Name | Sample Size | Mean | Variance |
| --- | --- | --- | --- |
| LS_2 | 7376 | 7.727 | 2.763 |
| LS_3 | 9063 | 7.556 | 2.804 |
| LS_4 | 7678 | 7.500 | 2.940 |
| LS_5 | 6961 | 7.543 | 2.637 |
| LS_6 | 6321 | 7.556 | 2.749 |
| LS_7 | 5703 | 7.521 | 2.738 |
| LS_8 | 5258 | 7.494 | 2.807 |
| LS_9 | 4935 | 7.553 | 2.497 |

Figure S1. *Cumulative Critical Life Events Frequencies Plot*

**
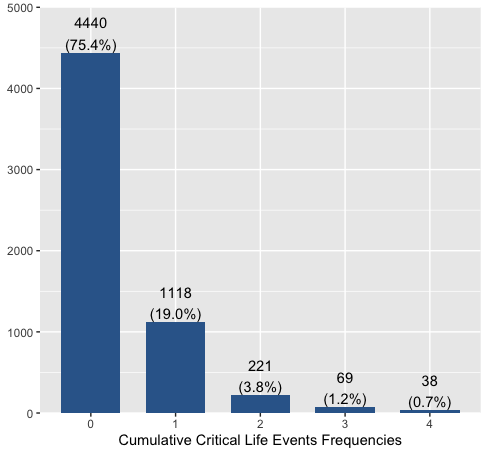
**

Table S4. *Fit Statistics for Life Satisfaction Single-Group (Nonmixture) Models*

| Model | CFI | TLI | RMSEA | *χ2* | df | *χ2/*df | AIC | BIC | Adjusted BIC | SRMR |
| --- | --- | --- | --- | --- | --- | --- | --- | --- | --- | --- |
| Intercept | 0.916 | 0.931 | 0.047 | 851.964*** | 34 | 25.058 | 199149.07 | 199221.97 | 199190.19 | 0.065 |
| Linear | 0.966 | 0.969 | 0.032 | 365.194*** | 31 | 11.780 | 198359.77 | 198454.53 | 198413.22 | 0.055 |
| Quadratic | 0.985 | 0.984 | 0.023 | 177.132*** | 27 | 6.560 | 198061.08 | 198185.01 | 198130.98 | 0.020 |
| Latent Basis | 0.969 | 0.965 | 0.034 | 329.867*** | 25 | 13.194 | 198266.56 | 198405.06 | 198344.68 | 0.051 |
| Cubic^a^ | N/A | N/A | N/A | N/A | N/A | N/A | N/A | N/A | N/A | N/A |

*Note*: N*s* = 10,824. CFI = Comparative Fit Index. TLI = Tucker-Lewis Index. RMSEA = Root Mean Square Error of Approximation. AIC = Akaike Information Criterion. BIC = Bayesian Information Criterion. SRMR = Standardized Root Mean Square Residual. ****p* < .001.

Table S5. *Fit Statistics for Life Satisfaction Quadratic LCGA*

|  | **1 Class** | **2 Classes** | **3 Classes** | **4 Classes** |
| --- | --- | --- | --- | --- |
| LL (NO. Of parameters) | -108789.66(11) | -102060.78(15) | -100008.58(19) | -99162.43(23) |
| AIC | 217601.32 | 204151.57 | 200055.16 | 198370.85 |
| BIC | 217681.50 | 204260.91 | 200193.66 | 198538.51 |
| Entropy | N/A | 0.818 | 0.694 | 0.687 |
| LRT-LMR test | N/A | 13105.07*** | 3935.27*** | 1647.96** |
| BLRT test | N/A | 13457.75*** | 3935.27*** | 1692.31*** |
| Group-size (%) Class 1 | 10824(100%) | 8871(82%) | 4219(39%) | 300(2.8%) |
| Class 2 | N/A | 1953(18%) | 786(7.3%) | 3188(29.5%) |
| Class 3 | N/A | N/A | 5819(53.7%) | 5861(54.1%) |
| Class 4 | N/A | N/A | N/A | 1475(13.6%) |

*Note:* LCGA = Latent Class Growth Model. LL = Log-Likelihood value. No. of Parameters = Number of estimated parameters. AIC = Akaike Information

Criterion. BIC = Bayesian Information Criterion. LMR-LRT = Lo-Mendell-Rubin Likelihood Ratio Test. BLRT = Bootstrap Likelihood Ratio Test.

***p* < .01. ****p* < .001.

Table S6. *Model Results of the Life Satisfaction Quadratic GMM-CI for the 3-class solution*

| *MODEL RESULTS* |  | ***Class 1*** |  |
| --- | --- | --- | --- |
|  |  |  | *Two Tailed* |
|  | *Estimate* | *S.E.* | *P- Value* |
| **Means** |  |  |  |
| Intercept | 6.328 | 0.241 | 0.000 |
| Slope | 0.091 | 0.162 | 0.575 |
| Quadratic term | -0.071 | 0.024 | 0.003 |
| **Variances** |  |  |  |
| Intercept | 0.761 | 0.059 | 0.000 |
| Slope | 0.133 | 0.016 | 0.000 |
| Quadratic term | 0.002 | 0.000 | 0.000 |
|  |  | ***Class 2*** |  |
| **Means** |  |  |  |
| Intercept | 3.732 | 0.196 | 0.000 |
| Slope | 1.061 | 0.155 | 0.000 |
| Quadratic term | -0.088 | 0.018 | 0.000 |
| **Variances** |  |  |  |
| Intercept | 0.761 | 0.059 | 0.000 |
| Slope | 0.133 | 0.016 | 0.000 |
| Quadratic term | 0.002 | 0.000 | 0.000 |
|  |  | ***Class 3*** |  |
| **Means** |  |  |  |
| Intercept | 8.017 | 0.022 | 0.000 |
| Slope | -0.166 | 0.012 | 0.000 |
| Quadratic term | 0.021 | 0.002 | 0.000 |
| **Variances** |  |  |  |
| Intercept | 0.761 | 0.059 | 0.000 |
| Slope | 0.133 | 0.016 | 0.000 |
| Quadratic term | 0.002 | 0.000 | 0.000 |

*Note*: GMM-CI = Growth Mixture Model with class-invariant variances and

covariances; S.E. = Standard Error.

Table S7. *Fit Statistics for Health Single-Group (Nonmixture) Models*

| Model | CFI | TLI | RMSEA | *χ2* | df | *χ2/*df | AIC | BIC | Adjusted BIC | SRMR |
| --- | --- | --- | --- | --- | --- | --- | --- | --- | --- | --- |
| Intercept | 0.949 | 0.958 | 0.036 | 516.378*** | 34 | 15.189 | 146361.21 | 146434.09 | 146402.32 | 0.039 |
| Linear | 0.982 | 0.984 | 0.022 | 198.103*** | 31 | 6.391 | 146000.84 | 146095.61 | 146054.29 | 0.026 |
| Quadratic | 0.989 | 0.989 | 0.019 | 130.536*** | 27 | 4.835 | 145931.03 | 146054.95 | 146000.93 | 0.017 |
| Latent Basis | 0.982 | 0.980 | 0.025 | 192.869*** | 25 | 7.715 | 145996.55 | 146135.05 | 146074.67 | 0.025 |
| Cubic^a^ | N/A | N/A | N/A | N/A | N/A | N/A | N/A | N/A | N/A | N/A |

*Note:* N*s* = 10,822. CFI = Comparative Fit Index. TLI = Tucker-Lewis Index. RMSEA = Root Mean Square Error of Approximation. AIC = Akaike Information Criterion. BIC = Bayesian Information Criteria. SRMR = Standardized Root Mean Square Residual. ****p* < .001.

Table S8. *Fit Statistics for Health LCGA*

|  | **1 Class** | **2 Classes** | **3 Classes** | **4 Classes** |
| --- | --- | --- | --- | --- |
| LL (NO. Of parameters) | -78874.69 (11) | -74047.29 (15) | -73138.36 (19) | -72871.87 (23) |
| AIC | 157771.39 | 148124.59 | 146314.72 | 145789.75 |
| BIC | 157851.57 | 148233.93 | 146453.22 | 145957.41 |
| Entropy | N/A | 0.695 | 0.614 | 0.574 |
| LRT-LMR test | N/A | 9401.78*** | 1770.22*** | 524.89*** |
| BLRT test | N/A | 9654.80*** | 1817.87*** | 524.89*** |
| Group-size (%) Class 1 | 10822 (100%) | 7539 (69.7%) | 4016 (37.1%) | 1740 (16.1%) |
| Class 2 | N/A | 3283 (30.3%) | 1156 (10.7%) | 6284 (58%) |
| Class 3 | N/A | N/A | 5650 (52.2%) | 2278 (21.1%) |
| Class 4 | N/A | N/A | N/A | 520 (4.8%) |

*Note:* LCGA = Latent Class Growth Model. LL = Log-Likelihood value. No. of Parameters = Number of estimated parameters. AIC = Akaike Information Criterion.

BIC = Bayesian Information Criterion. LMR-LRT = Lo-Mendell-Rubin Likelihood Ratio Test. BLRT = Bootstrap Likelihood Ratio Test. ****p* < .001.

Table S9. *Model Results of the Health Constrained Quadratic GMM-CI for the 4-class Solution*

| *MODEL RESULTS* | | ***Class 1*** |  |
| --- | --- | --- | --- |
|  |  |  | *Two Tailed* |
|  | *Estimate* | *S.E.* | *P- Value* |
| **Means** |  |  |  |
| Intercept | 2.475 | 0.050 | 0.000 |
| Slope | 0.103 | 0.037 | 0.006 |
| Quadratic term | -0.003 | 0.005 | 0.605 |
| **Variances** |  |  |  |
| Intercept | 0.129 | 0.008 | 0.000 |
| Slope | 0.000 | 0.000 | 999’000 |
| Quadratic term | 0.000 | 0.000 | 0.000 |
|  |  | ***Class 2*** |  |
| **Means** |  |  |  |
| Intercept | 2.359 | 0.047 | 0.000 |
| Slope | 0.761 | 0.060 | 0.000 |
| Quadratic term | -0.089 | 0.009 | 0.000 |
| **Variances** |  |  |  |
| Intercept | 0.129 | 0.008 | 0.000 |
| Slope | 0.000 | 0.000 | 999’000 |
| Quadratic term | 0.000 | 0.000 | 0.000 |
|  |  | ***Class 3*** |  |
| **Means** |  |  |  |
| Intercept | 3.984 | 0.069 | 0.000 |
| Slope | -0.697 | 0.055 | 0.000 |
| Quadratic term | 0.083 | 0.008 | 0.000 |
| **Variances** |  |  |  |
| Intercept | 0.129 | 0.008 | 0.000 |
| Slope | 0.000 | 0.000 | 999’000 |
| Quadratic term | 0.000 | 0.000 | 0.000 |
|  |  | ***Class 4*** |  |
| **Means** |  |  |  |
| Intercept | 4.141 | 0.015 | 0.000 |
| Slope | -0.081 | 0.009 | 0.000 |
| Quadratic term | 0.006 | 0.001 | 0.000 |
| **Variances** |  |  |  |
| Intercept | 0.129 | 0.008 | 0.000 |
| Slope | 0.000 | 0.000 | 999’000 |
| Quadratic term | 0.000 | 0.000 | 0.000 |

*Note*: GMM-CI = Growth Mixture Model with class-invariant variances and

covariances. S.E. = Standard Error.

**References**

1. Wickrama KAS, Lee TK, O’Neal CW, Lorenz FO. Higher-Order Growth Curves and Mixture Modeling with Mplus: A Practical Guide. New York: Routledge; 2016.

2. Infurna FJ, Luthar SS. Re-evaluating the notion that resilience is commonplace: A review and distillation of directions for future research, practice, and policy. Clin Psychol Rev [Internet]. 2018;65(July):43–56. Available from: https://doi.org/10.1016/j.cpr.2018.07.003

3. Grimm KJ, Ram N, Estabrook R. Growth Modeling: Structural Equation and Multilevel Modeling Approaches. Little TD, editor. New York: The Guildford Press; 2016.
